# Go with the flow: A neuroscientific view on being fully engaged

**DOI:** 10.1111/ejn.15014

**Published:** 2020-11-09

**Authors:** Dimitri van der Linden, Mattie Tops, Arnold B. Bakker

**Affiliations:** ^1^ Department of Psychology, Education, and Child Studies Erasmus University Rotterdam Rotterdam The Netherlands; ^2^ Developmental and Educational Psychology Unit Leiden University Leiden The Netherlands; ^3^ University of Johannesburg South Africa

**Keywords:** brain networks, flow, neuroscience of optimal performance, task engagement

## Abstract

Flow is a state of full task absorption, accompanied with a strong drive and low levels of self‐referential thinking. Flow is likely when there is a match between a person's skills and the task challenge. Despite its relevance for human performance and the vast body of research on flow, there is currently still relatively little insight in its underlying neurocognitive mechanisms. In this paper, we discuss a set of large brain networks that may be involved in establishing the core dimensions of flow. We propose that dopaminergic and noradrenergic systems mediate the intrinsic motivation and activate mood states that are typical for flow. The interaction between three large‐scale attentional networks, namely the Default Mode Network, Central Executive Network and the Salience Network is proposed to play a role in the strong task engagement, low self‐referential thinking, feedback and feelings of control in flow. The proposed relationships between flow and the brain networks may support the generation of new hypotheses and can guide future research in this field.

## Graphical abstract

Flow is the experience of full task engagement and low self‐referential thinking. It plays a relevant role in human performance and well‐being. We propose a neuroscientific model in which dopaminergic and norepinephrine networks underlie the motivational aspects of flow. The interplay between the default mode network, the salience network and the central executive network subsequently regulate the attentional properties of flow. The model may guide future neuroscientific research on flow.

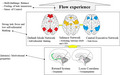

**Abbreviations**
ACC: Anterior cingulate cortex
AIC: Anterior insular cortex
CEN: Central executive network
DLPFC: Dorsolateral prefrontal cortex
DMN: Default mode network
EEG: Electroencephalography
LC‐NE system: Locus coeruleus‐norepinephrine system
MRI: Magnetic resonance imaging
Nacc: Nucleus accumbens
PPC: Posterior parietal cortex
SN: Salience network

## 1 INTRODUCTION

When working on a task, people sometimes enter a state that is characterized by being fully engaged, up to the point where they tend to have very low levels of self‐reflection and are hardly conscious of their surroundings. In the literature, this specific state of strong focus and immersion in the activity is often referred to as ‘flow’ (Csikszentmihalyi, 1988, 2014). The phenomenon of flow is well known and is frequently and anecdotally reported in relation to the performance of artists, athletes and scientists (Eisenberger et al., 2005). Yet, when the conditions are right, flow may also occur in everyday life when being involved in more daily challenging or interesting activities such as during work or leisure time (Bakker, 2008; Csikszentmihalyi, 2014; Demerouti et al., 2012; LeFevre, 1988). The concept of flow was introduced in the seminal work of Csikszentmihalyi (1975) who observed people working relentlessly on tasks without seemingly getting bothered by fatigue, boredom or other negative mood states or cognitions (e.g. self‐doubt).

Flow has been the subject of many studies and has become one of the cornerstones of positive psychology (Nakamura & Csikszentmihalyi, 2014). Many scholars consider flow as highly relevant to human performance in various domains (e.g. Demerouti, 2006; Eisenberger et al., 2005). One of the limitations in the field, however, is that flow has mainly been studied using self‐report measures. Although the experience of flow is, of course, subjective by nature, an overreliance on subjective (self‐report) methods limits the refinement of the construct and its divergent validity vis‐à‐vis other constructs such as motivation, or simply optimal task performance. For example, there is an ongoing discussion on the extent to which flow differs from other mental states such as mindfulness or strong concentration (Kee & Wang, 2008; Reid, 2011; Sheldon et al., 2015). In the present paper, we argue that in order to more fully understand the flow, it is useful to study the concept from a neuroscientific perspective. One of the advantages of doing so is that it forces one to be more precise in the conceptualization and operationalization of the dimensions of flow. In addition, a neuroscientific approach opens up possibilities for new insights into flow and possible novel ways of measuring the phenomenon.

In light of the above, we consider it timely and relevant to further theorize on the neuropsychological structures and functions that may be associated with flow. The present paper is by no means the only or the first to discuss flow from a neuroscience perspective. More than 15 years ago, Dietrich (2004) published a theoretical paper on how flow may work in the brain. Following this article, a handful of empirical studies have been carried out testing the neurological structures that are activated (or deactivated) during flow. More recently, the book chapter of Harris et al. (2017a) reviewed the neurocognitive mechanisms of flow during sports. The current paper, however, goes beyond the extant literature by incorporating new insights from neurocognitive research, and providing a more in‐depth discussion of flow in various life domains (e.g. not only sports, but also work‐related flow and gaming), the possible neurological structures involved and their functions. But, first we will provide a brief summary of the basic dimensions of flow.

## 2 CHARACTERISTICS OF FLOW

The literature reveals an ongoing effort to refine our understanding of flow and its main characteristics (Engeser, 2012). Several scholars have discussed the nine dimensions of flow that seem to be consistent in the literature (e.g. Csikszentmihalyi, 1975; Fullagar & Kelloway, 2009; Moneta, 2012). The first dimension is referred to as fusion of action and consciousness, implying that most of the cognitive processing is directed at the ongoing behaviour. Second, and in line with the first dimension, is the high level of focus or concentration that is typical of flow. Third, there is a reduction in self‐consciousness, which in this case implies low levels of self‐reflection and not worrying about what others would be thinking of oneself. The fourth characteristic is the feeling of being in control. Fifth, individuals experiencing flow have clear goals. The sixth dimension is feedback, because during flow one often ‘knows’ how performance is going. Autotelic experience is the seventh dimension and implies that the experience of flow in itself is pleasant and/or rewarding and yields a tendency or desire to experience that state again. The eighth dimension involves a changed subjective experience of time—time often seems to fly when being in flow, which has been confirmed in a recent meta‐analysis of Hancock et al. (2019). The ninth dimension is the experienced balance between the level of challenge or task difficulty on the one hand, and one's skill level on the other hand (Keller et al., 2011). This latter dimension turned out be one of the key dimensions of flow because when a task is very easy, it is rather unlikely that one will experience flow. Instead, feelings of boredom and mind‐wandering may occur. Also, when the task becomes too demanding or too difficult, it is likely that one will experience stress and a lowered sense of control (Keller, 2016). Thus, boredom as well as stress tend to disrupt any experience of flow. An intermediate level of arousal, however, seems to be optimal for experiencing flow (Peifer et al., 2014; Tozman et al., 2015). The nine basic dimensions of flow as described above are also summarized in Table 1.

**Table 1:** The nine most basic flow experience characteristics

Flow characteristic	Description
1. Fusion of action and consciousness	Several aspects of the task are executed in ‘an automatic’ way
2. High Focus/concentration	The person being fully engaged into the task at hand
3. Reduced self‐reflection/absence of worrying/forgetting environment	Probably due to the focus, levels of thinking about oneself and worrying are low and non‐task‐relevant aspects of the environment are ignored.
4. Being in control	The person has the idea that adequate performance can be maintained
5. Clear goals	The person knows what has to be done and what aims to achieve.
6. Feedback	There is an ongoing (not necessary conscious) monitoring of performance.
7. Autotelic property	The experience has rewarding properties (has some addictive elements)
8. Changed experience of time	Subjective time passes by relatively quickly
9. Balance between skills and task challenge	The person's knowledge and skills allow optimal performance (at a personal standard)

One rather salient aspect of flow that, remarkably, does not seem to be included in the list of basic dimensions of flow is the high level of dedication, energy or vigour associated with flow (Csikszentmihalyi, 2014). Think, for example, of the gamer or computer programmer, engaging in their tasks relentlessly for extended periods of time. Another aspect of flow that was not mentioned yet is mood state. On this topic, there seems to be less consensus. Several scholars have argued that flow is, by definition, accompanied with enjoyment or positive mood states (e.g. Eisenberger et al., 2005). However, it remains unclear whether one necessarily has to experience enjoyment during the flow state. As we will also refer to in this paper, it is more likely that flow is particularly associated with activating mood states—mood that enhances energy, drive, and persistence and that includes positive mood states such as optimism or pleasure, but also negative mood states such as anger.

Whether the flow characteristics outlined above are basic, or rather manifestations of an even more general process remains an open question. For example, the feeling of being in control and knowing what to do and what goals to achieve possibly can be considered the subjective equivalents of the balance between one's level of skill and the task challenge. Yet, going into the details of the various dimensions of flow as emphasized in the literature and their relative contribution is beyond the scope of the present paper.

Any neuroscientific conceptualization of flow, however, should take into account how several of these separate dimensions of flow are established. For example, a neuroscientific conceptualization should explain how flow is accompanied with reduced introspection or thinking about oneself. Also, the strong investment into a set of activities during flow suggests that conscious or unconscious decisions are made to strongly engage in one type of behaviour at the cost of neglecting other behavioural options. Thus, a neuroscientific account of flow should also incorporate ideas on how the brain ‘decides’ whether to continue with the current task versus switching to other options.

In the present review, we will use the flow dimensions outlined above when discussing various well‐known brain systems. The first two brain systems we discuss are neuromodulatory systems driving task motivation or task engagement. Subsequently, we will elaborate on three large brain systems that are affected by the neuromodulatory systems and that play a role in the attentional regulation in task focus and self‐reflection.

## 3 DEDICATION, PERSISTENCE AND MOOD DURING FLOW: THE POSSIBLE INVOLVEMENT OF TWO NEUROMODULATORY SYSTEMS

Although high concentration, low self‐reflection and forgetting of one's surroundings may be critical aspects of flow (Moneta, 2012), we wish to emphasize that before such a state occurs, at some point, one has already made the decision that the task is motivating or relevant enough to fully engage in it in the first place. Thus, the task either has to be intrinsically rewarding or has to be in accordance with the (short‐ or long term) goals one wants to achieve (Abuhamdeh & Csikszentmihalyi, 2012; Keller & Bless, 2008). For example, for academics, engaging in writing or conducting data analyses will more likely lead to flow, compared to engaging in unavoidable administrative work. In principle, there is nothing intrinsic in doing administration that would prevent one from experiencing flow; however, for scientists (and probably for many other people), engaging in it would simply distract from reaching their main and desired goals. In contrast, for administrators or accountants, administrative work may be central to their main occupational goals. They may also really enjoy such tasks and therefore, administrative tasks may more likely yield flow in this occupational group. More generally, if the conditions of intrinsic motivation or high goal relevance are not met, then flow is unlikely. As also mentioned before, flow does not tend to occur in tasks that are deemed boring, useless or overly stressful (Keller et al., 2011; Peifer et al., 2014).

In addition, the literature suggests that flow is accompanied with mood states that are activating and supportive of task engagement and goal‐directed (approach) behaviours and mindsets (Bloch, 2002; Fullagar & Kelloway, 2009). Examples of such mood states are enjoyment, hope (i.e. expectation of success), energetic drive and anger. Deactivating mood states, such as stress, fear and perceived helplessness, on the other hand, tend to inhibit action and may therefore also disrupt subjective and behavioural manifestations of flow (Van der Linden et al., 2007).

Two neuromodulatory systems that may be involved in establishing the motivating dimensions of flow are the brain's reward systems (Ikemoto & Panksepp, 1999) and the locus coeruleus‐norepinephrine system, or LC‐NE system (Aston‐Jones & Cohen, 2005; Benarroch, 2009). The former has mainly been linked to flow proneness in a few previous articles (De Manzano et al., 2013; Niksirat et al., 2019). However, to the best of our knowledge, there is no literature yet that has explicitly linked the LC‐NE system to flow, in a theoretical or empirical way.

### 3.1 Motivation and mood during flow: The brain's dopaminergic reward systems

Artist, athletes or experts on a specific topic may relatively often experience flow because they tend to do the things they consider highly and intrinsically rewarding (Abuhamdeh & Csikszentmihalyi, 2012; Keller & Bless, 2008). Several subcortical brain areas are known to be involved in mediating the rewarding aspects of activities (Robbins & Everitt, 1996). For example, the *nucleus accumbens* is a well‐studied structure in the so‐called cortico‐basal ganglia‐thalamo‐cortical loop (Ikemoto & Panksepp, 1999). The nucleus accumbens receives dopaminergic input from the ventral tegmental area, located in the mid‐brain (Salamone, 1994). Dopamine is a neurotransmitter that plays a crucial role in establishing the rewarding and reinforcing aspects of behaviour (Wise, 2004). The nucleus accumbens is often considered a core component of the brain's dopaminergic reward system. Importantly, the activation of the nucleus accumbens and the accompanying dopaminergic pathways tend to coincide with the experience of mainly activating emotions such as enjoyment, hope, optimism and craving (Buckholtz et al., 2010; Salamone, 1994). This is similar to the feelings that have been associated with flow (Csikszentmihalyi, 2014).

In neuroscience, a distinction is typically made between the nucleus accumbens outer area, the shell (NAcc Shell), versus its inner area, the core (NAcc Core; Di Chiara et al., 2004). This is partly related to the functional distinction between *liking* versus *wanting* (Berridge et al., 2009; Salamone et al., 2007). *Liking* refers to the level of enjoyment that is experienced when rewards are obtained (e.g. getting food or engaging in pleasurable activities). The NAcc Shell plays a relevant role in this. *Wanting*, however, refers to the level of craving or energetic drive one has in trying to obtain a certain reward (Berridge et al., 2009). The NAcc Core predominantly affects the ‘wanting’ as it plays a particularly relevant role in the cognitive processes and motor functions directed at obtaining the reward. In general, wanting relates to the willingness for effort expenditure directed at achieving some desired outcome (Salamone et al., 2009). The literature also clearly indicates that the NAcc as well as other structures within the dopaminergic systems are involved in weighing the effort it would take to achieve a goal against the level of reward it provides (Boksem & Tops, 2008; Salamone et al., 2018).

Given the characteristics of the brain's reward system, it seems reasonable to assign a role of this system to the experience of flow. Research that has explicitly tested this notion is scarce, but there is, nevertheless, some empirical support for it. De Manzano et al. (2013) showed that individual differences in the proneness to experience flow are related to higher availability of dopamine D2 receptors in the striatum of which, among others, the nucleus accumbens is a substructure. Dopaminergic activity in the striatum mediates reward processing. Moreover, individual differences in the D2 receptor density and availability have been linked to traits that have shown to relate to flow, such as low impulsivity and high emotion regulation abilities (Blasi et al., 2009). Although flow proneness is not the same as the actual experience of flow, the fact that dopamine pathways have been found to play a role in flow proneness seems to suggest that they may also be involved in the subjective experience of flow itself.

Other more direct, empirical support for the role of the reward systems in flow experience comes from the studies of Ulrich et al. (2014), Ulrich et al. (2016) who used a within‐person design in which participants were tested (they had to do calculations) in a brain scanner (i.e. MRI) during three different conditions—a boredom condition, in which the task was relatively easy, an overload condition, in which the task was too difficult for the participants' skill levels and a condition that was assumed to induce flow due to the matching of task difficulty and participant's skill level. The flow condition was related to significantly increased activity in the basal ganglia, including the nucleus accumbens. Obviously, the task that Ulrich et al. used was very specific (i.e. mental calculations) and possibly cannot be directly generalized to other flow‐inducing activities such as sports activities, playing music or creating art. Yet, based on the idea that flow can occur in a wide range of tasks in which skills and challenges are matched, Ulrich et al.'s findings may be indicative of what largely happens in the brain during flow in general.

Additional, albeit more indirect, support for the role of dopaminergic systems comes from a study in which the tendency to be fully absorbed in their work (which resembles flow‐like states) was related to individual differences in markers of the sensitivity of the behavioural reward system (Van der Linden et al., 2007).

Conceptually, a direct link between flow and the brain reward systems seems to make sense (Weber et al., 2009). Tasks that have the potential to activate reward systems will ‘energize’ behaviour directed at that task and is accompanied with a strong task engagement and intrinsic motivation (Berridge et al., 2009). At this point, it is relevant to mention that once the reward systems are very active, they also tend to diminish feelings of fatigue, pain or other subjective discomfort. For example, ample research has confirmed that substances that directly or indirectly enhance the brain dopamine's levels, such as coffee or amphetamine, also tend to counter fatigue (Stahl, 2002). Those properties of the brain's dopaminergic pathways fit nicely with observations that people in a state of flow can work relentlessly for a considerable amount of time without feeling or showing clear signs of discomfort, resistance (e.g. experienced as excessive effortfulness) or fatigue (Csikszentmihalyi, 1988), as any parent with a teenage gamer at home may easily acknowledge.

Finally, stimuli or activities that can activate reward systems usually also reinforce the desire to experience that same state again (Di Chiara, 2002), which to some extent is similar to how an addiction works (although it may be less intense). This aligns with the idea of flow as an *autotelic experience*—a state that is pleasurable in itself and leads to the desire to experience that same state again in the future (Fullagar & Kelloway, 2009)—which suggests that indeed some level of reward reinforcement, mediated by the brain reward systems, seems to occur in flow.

The notion that the brain's reward systems are involved in flow may contribute to more fundamental and new insights into the nature of the construct, and may also generate new research questions. To illustrate, many previous studies reported positive mood associated with flow (Bloch, 2002; Csikszentmihalyi, 2014; Eisenberger et al., 2005). However, given that this was mainly established using surveys that participants filled in after they experienced flow, those findings may particularly relate to the *liking* aspect of reward—it may show how satisfied/happy people feel with the reward they have already received from the flow. However, during the actual flow experience, the state may be particularly associated with the *wanting* aspect of motivation. This aspect more strongly relates to activation and drive instead of satisfaction (Berridge et al., 2009; Salamone et al., 2009). In line with this, it would be useful if future research would investigate the possible differential roles of wanting versus liking in flow and their associated neurological structures (e.g. NAcc core versus NAcc shell).

### 3.2 Continuing or stopping? A role of the locus‐coeruleus norepinephrine system in flow?

Traditionally, dopaminergic reward systems are presumed to play a role in the direction of actions—that is, *which* activities to focus on—and in decisions on whether or not to continue with the current line of actions, based on the trade‐off between the costs and rewards of actions (Boksem & Tops, 2008; Ikemoto & Panksepp, 1999). More recently, however, scholars have also emphasized the possible role of the locus *coeruleus‐norepinephrine* (LC‐NE) system in such decisions (Aston‐Jones & Cohen, 2005). The LC is a small nucleus in the pons of the brain and is the principle site of central norepinephrine (noradrenaline) release (Benarroch, 2009). The LC has widespread connections to other brain areas such as the amygdala and hippocampus, the cerebellum, the cerebral cortex and also the ventral tegmental area, which we identified in the previous section as the origin of dopamine cells that feed into other reward‐related structures (Ranjbar‐Slamloo & Fazlali, 2020).

In the earlier literature, it was assumed that the LC‐NE system mainly had the relatively simple function of regulating the brain's arousal levels (e.g. Robinson & Berridge, 1993). It plays a significant role in sleep and wakefulness. However, it has now been established that the LC‐NE system has more elaborate functions involving motivation and attention. In their seminal review, Aston‐Jones and Cohen (2005) highlighted the role of the LC‐NE system in task engagement. As such, a link with flow is plausible because a very strong task engagement is seen as one of the most salient characteristics of flow (Bakker, 2008). In fact, as we will explain below, there are various characteristics of the LC‐NE system that overlap quite well with important features of flow.

One presumed key function of the LC‐NE system is to support decisions on whether to maintain focus on the task at hand, or switch attention to other task and stimuli (Cohen et al., 2007). Such decisions are based on the balance between the current or future rewards involved in a set of activities versus its cost, such as resource depletion. If the balance is in favour of the rewards, one will maintain task focus. If the costs start to outweigh the rewards, then one tends to withdraw from the task (Boksem & Tops, 2008; Kurzban et al., 2013). Several empirical studies indeed support the idea that the LC‐NE system plays a pivotal role in the balancing between engagement versus disengagement (Aston‐Jones et al., 2000). It would go beyond the scope of the present review to provide a detailed account of how the LC‐NE system underlies such decisions. Essentially, the idea is that the LC‐NE system may regulate an attentional filter, based on configurations of tonic (baseline) NE release versus phasic (stimulus‐driven) NE release (Aston‐Jones et al., 2000). If a task is deemed rewarding enough, then the LC‐NE system produces a moderate level of baseline NE, with relatively strong stimulus‐driven pulses of NE release. In this configuration, a person has a sufficient level of arousal (i.e. is alert enough) to engage in the task, and also is highly attentive to task‐related events. Moreover, responses to non‐task‐relevant stimuli are low, implying low distraction. Aston‐Jones and Cohen (2005) refer to this set of behavioural characteristics as the ‘task engagement mode’. The overlap with flow may be apparent because flow is indeed characterized by a strong task engagement, persistence (i.e. continuous exploitation) and the neglect of almost everything that is not task related (Csikszentmihalyi, 1990). If the reward/cost balance becomes unfavourable, the LC‐NE system changes its NE output such that task disengagement is likely.

**Figure 1:**
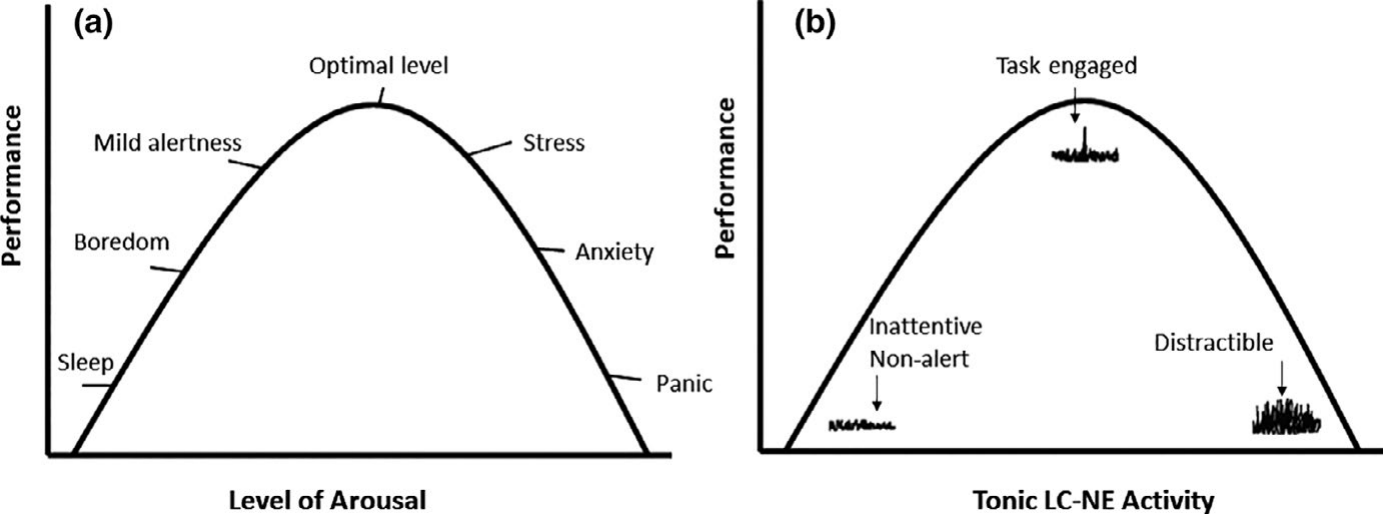
(a) Inverted U‐curve of performance and arousal. (b) The Inverted U‐curve in terms of tonic NE and pulses of NE to task relevant and irrelevant stimuli

There are two different ways in which the LC‐NE can mediate task disengagement, which are associated with two different task modes (see Figure 1). First, it can lower the baseline NE output as well as phasic NE responses. This results in a ‘general inattentiveness mode’ (Hopstaken et al., 2015). The second option is that it increases the baseline NE output but also the responsiveness of phasic NE release. This implies that general arousal is high and one tends to respond to task‐relevant as well as task‐irrelevant stimuli. This results in an overall ‘distractiveness mode’, which can also be described as a tendency to explore the environment for more interesting options than the current activities.

Regarding the LC‐NE system's role in balancing engagement versus disengagement, there is a set of empirical findings that fits well with one of the key dimensions of flow, namely the balance between skills and challenge (e.g. Nieuwenhuis et al., 2005). That is, in the LC‐NE system, the relevance of the skill/challenge match has been studied by systematically balancing the levels of effort for different levels of reward (Aston‐Jones & Cohen, 2005). In doing so, it has been found that situations in which the task difficulty (or task challenge) is high, but still doable, the LC‐NE system tends to go into the ‘task engagement mode’ (depicted in Figure 1). Yet, at some point, the task becomes so difficult that further investment in it seems less useful, even though the potential reward (e.g. the monetary incentive) is higher. Under that condition, the LC‐NE system changes its output to higher baseline NE and a more general phasic NE responsiveness. This implies the task disengagement and/or distraction modes.

To the best of our knowledge, the previous literature has not made an explicit link between flow and the LC‐NE system. We, nevertheless, argue that such a link has considerable potential as one of the underlying neurocognitive mechanisms regulating flow, particularly because it is typically discussed in the context of task engagement. Note that, the inverted U‐curve of the LC‐NE system, with its boredom, engagement and stress/distraction modes as depicted in Figure 1, seems highly similar to the inverted U‐curve of arousal and flow as described by Peifer et al. (2014). This overlap is in line with the hypothesis that the LC‐NE system is indeed involved in flow. Any contribution of the LC‐NE system to flow would obviously not be in isolation, but rather in collaboration with various other brain systems and structures. It is relevant to note here that various dopamine systems have descending connections to the LC‐NE system (Ranjbar‐Slamloo & Fazlali, 2020). Dopamine is also a precursor of NE. In terms of the psychological processes involved, dopaminergic systems may serve as information on the task's rewarding aspects, after which the LC‐NE system can switch to, or stay in, an engage or disengage mode. In the current context, this would suggest that the two brain systems (i.e. reward system and the LC‐NE) may play a role in initiating, maintaining and disrupting a state of flow (see also below).

### 3.3 From neuromodulatory systems to further cognitive processing in flow: Large‐scale attentional brain networks

In the previous sections, we focused on how dopaminergic and noradrenergic systems may underlie the motivation, mood states and task engagement in flow. We analysed how various dimensions of flow, such as intrinsic motivation, focus and high energy, may be intrinsically linked to the established characteristics of those systems. Yet, in order to more fully understand the wide range of behavioural and subjective facets of flow, it would be useful to take another subset of large‐scale brain networks into account—a subset that is assumed to regulate attention and may be connected to flow. Specifically, the networks we will further discuss have been theoretically linked to various cognitive processes such as controlled versus automatic cognitive processing and self‐awareness.

## 4 LARGE‐SCALE NETWORKS INVOLVED IN FLOW

Trying to explain a relatively broad phenomenon, such as flow, in terms of its underlying brain structures and functions can be considered an extensive challenge by any means. The brain is an incredibly complex organ with numerous specific areas that have distinct—and often not yet fully understood—functions. Moreover, many of the brain's functions are established by connections and interactions between various areas. Therefore, it is unlikely that a multifaceted state, such as flow, would be linked to a discrete number of brain areas or functions. A more useful approach is to discuss the topic in terms of several of the identified, so‐called, larger‐scale attentional brain networks (Bressler & Menon, 2010). These are sets of brain areas that are known to have close interconnections, are often activated (or de‐activated) in tandem and that are ascribed relatively broad functionalities. Often those systems receive input from and are regulated by the same neuromodulatory (DA and NE) systems we have already discussed above.

Here, we will focus on three of those systems, namely the Default Mode Network (DMN), the Central Executive Network (CEN) and the Salience Network (SN). For each network, we will start with a short general description of its nature and function, followed by its proposed relation to flow.

### 4.1 The Default Mode Network: Reduced self‐awareness and feedback in flow?

#### 4.1.1 Basic characteristics of the DMN

**Figure 2:**
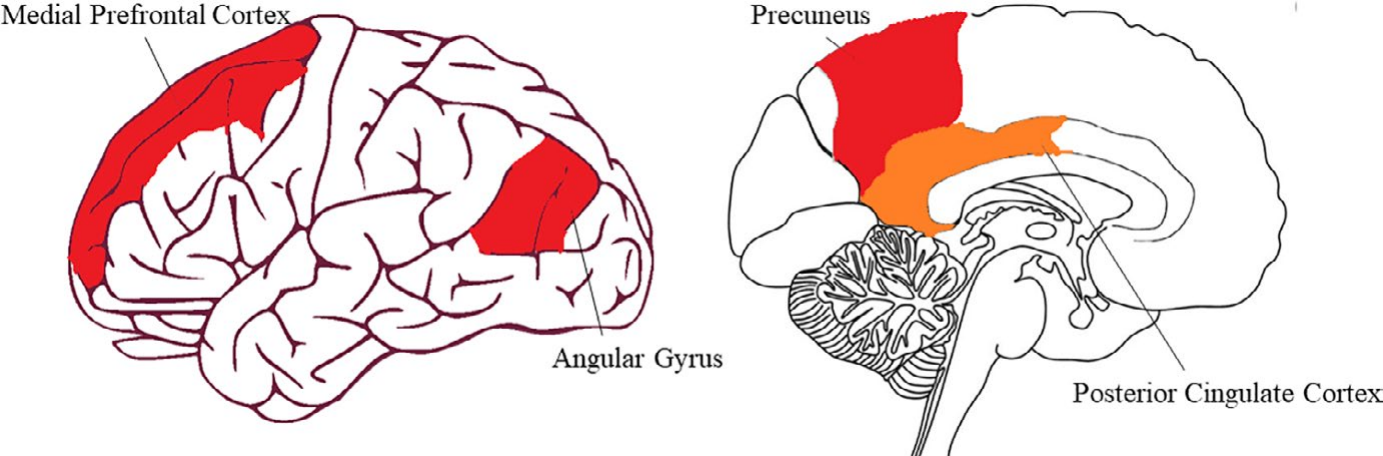
The main brain structures of the default mode network

Early brain imagining studies have discovered that during periods when participants are not engaged in cognitive or other external tasks, certain brain areas tend to become *more* activated (e.g. Shulman et al., 1997). This network of areas was labelled the *Default Mode Network* (DMN: Buckner et al., 2008; Raichle et al., 2001). The largest brain areas associated with the DMN are the posterior cingulate cortex and precuneus, the medial prefrontal cortex (mPFC) and the angular gyrus (AG), see also Figure 2.

Two of the main aspects of the DMN are assumed to be self‐referential processing and autobiographic memory (Davey et al., 2016; Gusnard et al., 2001). It is particularly active when one is thinking about oneself, in past, current or future situations (e.g. anticipating outcomes, planning). As such, the DMN has also been connected to mind‐wandering (Christoff et al., 2009; Kucyi & Davis, 2014). Such mind‐wandering or ruminations should be considered as specific instances of the general function of the DMN to cognitively simulate future scenarios (Buckner & Carroll, 2007; Tops et al., 2014).

Decreased activation of the DMN during task engagement may be a sign of reduced mind‐wandering. In light of the foregoing, findings of increased activation of the DMN during social activities (Schilbach et al., 2008) also make sense because in those situations, thinking about oneself and possible outcomes of one's behaviour are relevant for adequate social performance (e.g. what is the potential impact of the things I am saying?).

Although there is some debate about the structure and nature of the DMN, and whether or not it is active in task processing (e.g. Elton & Gao, 2015), many studies have confirmed the above‐explained link between the DMN and ‘inward directed’ processing or self‐referential thinking (Davey et al., 2016).

#### 4.1.2 The DMN and flow

Given the general characteristics of the DMN, several propositions can be formulated about how this network may relate to the subjective experience of flow. The most obvious relation is that a reduction in worries or thinking about oneself (in negative *or* positive sense) during flow seems to implicate reduced DMN activity. Earlier neuroimaging studies confirmed this to some extent. Ulrich et al. (2014), Ulrich et al. (2016) conducted several laboratory tests in which participants were scanned in an MRI while working on task conditions that either induced boredom, flow or stress (also note the overlap with the three modes of the LC‐NE system discussed in Section 3.2). The mPFC, an important component of the DMN, showed an inverted U‐shaped pattern in which activation was lowest in the flow condition (and highest in the boredom condition).

A proposition we wish to make in this paper is that the lowered activation of the DMN during flow is established through, at least, two different pathways. The first one is that reduced negative stress (in which one has feelings and ideas of losing control) and activating mood states during flow ensure that the DMN does not become overly active. This idea will be outlined in the present section. The second pathway is that the high task focus that is characteristic of the absorption component of flow may take away processing time or resources from the DMN. This notion is discussed in the next section, when we also elaborate on the role of another network, namely the CEN in flow.

Regarding the first pathway, interestingly, in their flow condition, Ulrich et al. (2014) also found lowered activation of the amygdala. This structure is not a direct component of the DMN, yet is assumed to be strongly causally linked to its activation (Sylvester et al., 2020). The amygdala reacts vigorously to threatening situations, thereby inducing thoughts about possible future negative consequences for the self. Experiencing threat or being fearful of negative outcomes stands in sharp contrast to the experience of flow. This has been confirmed by psychophysiological studies showing that flow is associated with low sympathetic activity of the autonomic nervous system, suggesting low stress levels (Harmat et al., 2015). When one is performing a task on which one's capabilities are up to the challenge, then one often has the feeling of being in control of the situation, or making progress towards one's goals. However, if signs appear that one can no longer adequately deal with the task requirements (e.g. it becomes too difficult), then the possibility of not obtaining goals becomes a reality and sympathetic activity increases, likely induced by, among others, the LC‐NE system (Aston‐Jones & Cohen, 2005).

Once the idea of losing control starts to develop, any experience of flow will get disrupted. This is accompanied by a reduction in task focus and increased distractibility (see also the section on LC‐NE). The task disengagement, accompanied with the higher arousal, will probably induce self‐reflection and in that case, the DMN may become more active again. Although, in principle, such reflection can be positive or negative, in case of a task that is too difficult, the reflection often becomes manifest in an increase in worrying.

The hypothesis that the DMN is least active when in flow compared to working on boring or too difficult tasks was also recently supported in one of our own studies (Blinded For Review), in which we tested EEG power patterns during three conditions—a boring condition, a flow condition and an overly difficult condition. Particularly, electroencephalogram (EEG) measures in the 8–12 Hz frequency domain, that is, alpha power, suggested a pattern in which the activity was low in the flow condition. Several scholars have convincingly argued and shown that alpha power partly reflects activity of the DMN (Knyazev, 2013; Knyazev et al., 2012).

### 4.2 The central executive network: Focusing on the task

#### 4.2.1 Basic characteristics of the CEN

In some regards, the CEN (D'esposito et al., 1995) is the opposite of the DMN. The CEN consists of an array of strongly interconnected brain areas that are mainly active when engaging in ‘external’ cognitive processing (Bressler & Menon, 2010). That is, when one is engaged in tasks that require the active maintenance of information (or task set) in working memory, a switching between task requirements (i.e. switching task set) and the inhibition of irrelevant information (D'esposito et al., 1995; Menon & Uddin, 2010). In other words, the CEN becomes activated in situations that require focus or concentration. Notably, the CEN and the DMN often show contrasting patterns of activation. If the CEN becomes more active, the DMN decreases in activation, and vice versa (Chen et al., 2013).

**Figure 3:**
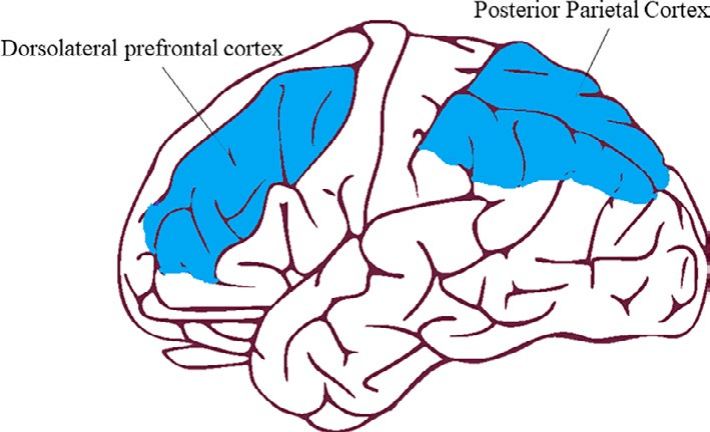
The main brain structures of the central executive network

The main brain areas associated with the CEN are the dorsolateral prefrontal cortex (DLPFC) and the posterior parietal cortex (PPC), see Figure 3 (Toro et al., 2008). Based on a vast amount of empirical evidence, the DLPFC has been referred to as the seat of working memory (e.g. Mars & Grol, 2007). Furthermore, the strength of the pathways between the DLPFC and PPC has been associated with intelligence—the ability to effectively deal with complex or novel problems and situations (Haier, 2016).

#### 4.2.2 The CEN and flow

In the literature, it has been suggested that reduced self‐awareness in flow may partly be due to competition for the brain's processing resources (e.g. processing time; Dietrich, 2004). This refers to the second pathway that we mentioned in the previous section. The competition for processing resources takes place between attention focused on external, task‐related stimuli versus self‐referential thinking (Sridharan et al., 2008). In this sense, it has much overlap with the above‐described oscillations between the DMN and the CEN. Stated differently, if one is busy focusing on the task at hand, there simply may be too few processing resources available to engage in reflection upon oneself.

This topic is intertwined with the fundamental question of what type of cognitive processes occurs during flow in the first place. The answer to that question is complex and introduces topics such as automatic versus controlled processing, and whether or not flow is associated with so‐called hypofrontality, that is, lowered activation of (certain parts of) the frontal lobes. Dietrich (2004) described information processing during flow in terms of explicit versus implicit processing. Explicit processing refers to controlled processing that includes the involvement of working memory, focused attention and conscious awareness of one's action. This type of processing is associated with CEN activity. Implicit processing refers to more automatic processing in which one applies previously (well) learned knowledge or skills that require little guidance of conscious attention.

The hypofrontality explanation of flow (Dietrich, 2004) suggests that during flow one mainly relies on implicit or automatic processing. This means that well‐learned behavioural or cognitive procedures smoothly follow each other, without much interference of conscious thinking. In several situations, in which flow occurs, this is indeed what seems to happen. Illustrative examples are highly skilled athletes, dancers or musicians who perform the acts that they have practiced intensively for so many times (e.g. Leroy & Cheron, 2020). Nevertheless, it is unlikely that flow is only related to the execution of well‐learned behavioural or cognitive sequences because in that case it can be expected to occur only in routine tasks, which is not the case. Specifically, flow often occurs during periods in which one can apply automatic behaviour in combination with a high enough challenge that requires a strong attentional focus. The latter implies that controlled processing is also involved (Harris et al., 2017b).

The empirical evidence seems to be in accordance with the idea that flow is indeed associated with a certain level of controlled processing. Although initial studies suggested that flow is associated with reduced frontal activity leading to the hypofrontal hypothesis (Dietrich, 2004), more recent studies have been unable to replicate this (Ulrich et al., 2014, although see, Leroy & Cheron, 2020). In fact, stronger activation of various frontal brain areas during flow conditions was found (Ulrich et al., 2014). In order to understand this, it may be useful to emphasize the distinction between frontal areas related to the CEN versus those that mainly relate to the DMN. The mPFC, for example, is part of the DMN which, in line with the previous discussion, can be expected to be less active during flow. Other frontal areas on the other hand, such as the DLPFC, as part of the CEN may be more active, as they play a role in keeping the task set active in mind and preventing its disruption by irrelevant information.

Another proposition we wish to pose, and that to our knowledge has not been directly empirically tested yet, is that the type of processing and brain activation during flow interacts with the type of task. Specifically, during some tasks, flow will mainly involve effortless, automatic processing and behaviour, whereas during other tasks, flow is associated with high concentration/effort and therefore high levels of controlled processing (Harris et al., 2017a, 2017b). To illustrate this, one may consider an athlete who engages in long‐distance running. At some point, he or she may feel all is going well and gets into the so‐called ‘runner's high’, which has also been associated with flow (Stoll, 2019). It can be expected that DMN as well as CEN activity would both be low and hypofrontality could be observed. Yet, it is known that this sensation of runner's high is related to the release of endorphins that suppress pain, fatigue and negative emotions (Boecker et al., 2008). In this regard, it may be a type of flow that is different from other task‐related flow. For example, in tasks in which individuals get fully engaged in cognitive activities, such as playing a challenging game, engaging in an important chess competition, solving a complex computer programming problem or writing a scientific paper, flow may be characterized by low DMN activity, possibly combined with strong CEN activation.

Such a distinction between the types of tasks can possibly also explain some of the contradictory findings in neuroscientific flow research. For example, in the fMRI studies of Ulrich et al. (2014) and Ulrich et al. (2016), no clear evidence was found for hypofrontality. However, in their studies they induced flow with solving math problems. This method, in real life, would more closely resemble tasks such as chess/programming/science, compared to the runner's high or the absorbed artist. Subsequently, it may not be that surprising that, in Ulrich et al.'s study, specific brain areas related to strong attentional focus and controlled processing (i.e. CEN components) were found to be more active during flow. In contrast to the studies of Ulrich et al. is the more recent study of Leroy and Cheron (2020), who studied brain activity in a professional tightrope performer. Their results were more in line with Dietrich's hypofrontality hypothesis. However, the task they examined was a clear well‐practiced movement task, and the flow that occurs during such a task may be more similar to the flow during sport performance than during complex problem‐solving. Accordingly, the possibility that the type of processing during flow is partly dependent on the type of task is a topic that certainly requires further scrutiny.

### 4.3 The Salience Network: Am I still on track?

#### 4.3.1 Basic characteristics of the SN

**Figure 4:**
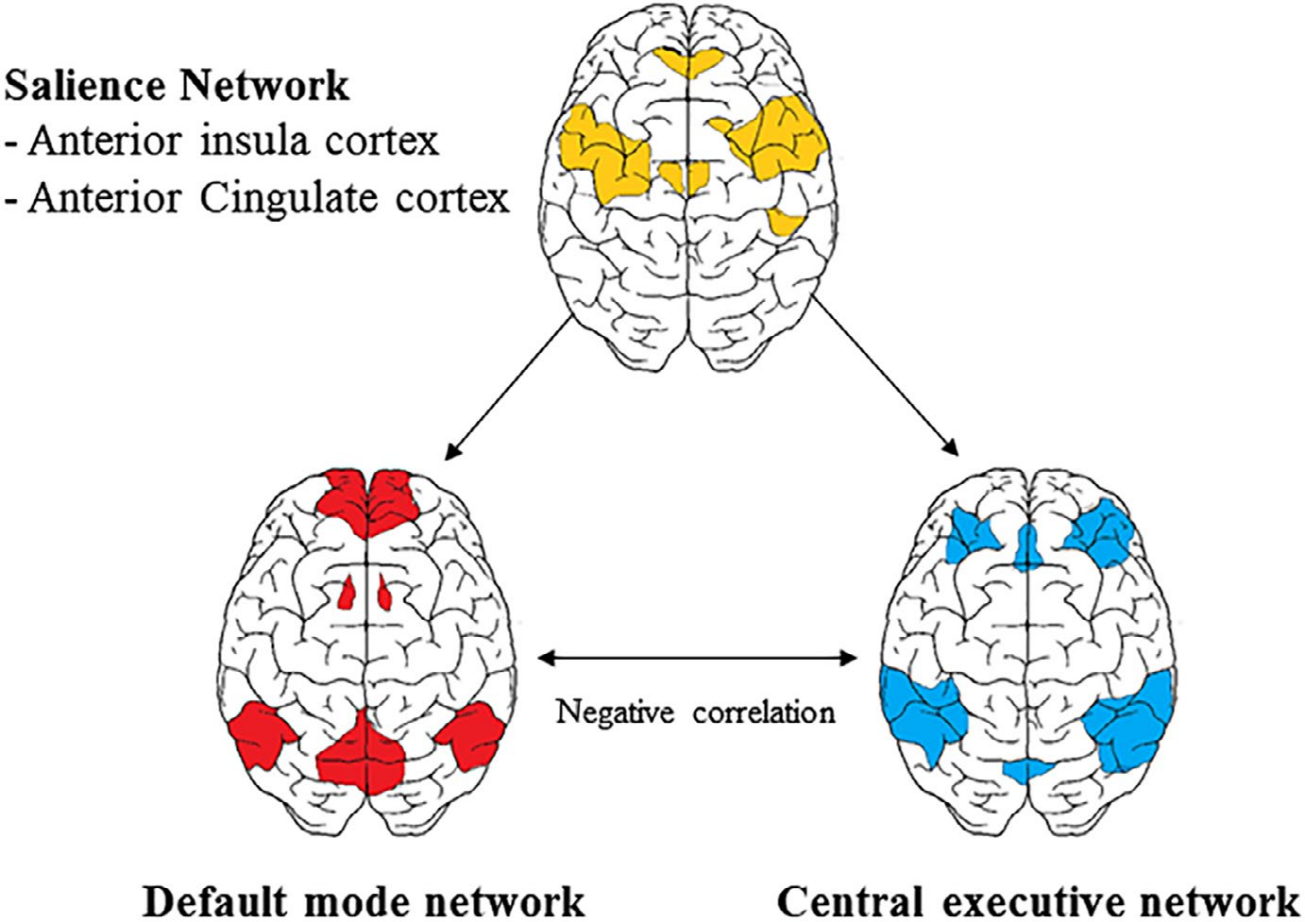
The salience network as the switch between activation of the default mode network (DMN) and central executive network (CEN)

The third, and final, network we consider in the neuroscientific perspective on flow is the so‐called SN (Menon & Uddin, 2010; Seeley et al., 2007). The main brain regions of this network are the anterior insula cortex (AIC) and the anterior cingulate cortex (ACC). Those two regions are also mentioned as the ones that are consistently active in almost all cognitive demands or tasks (Menon, 2015). Thus, they likely serve very broad functions. In recent theories, the SN has been ascribed the general role of switching between other brain networks (see Figure 4), particularly between the previously discussed DMN and the CEN (Sridharan et al., 2008). Accordingly, the SN is involved in the continuous switching between task‐related versus non‐task‐related and self‐referential processing. Or in more mundane terms, this switching may be related, but is not restricted, to the switching between task concentration and mind‐wandering. The network received its name due to its presumed core function, which is detecting the salience of stimuli/events. Salience, in this context, is every stimulus, internal or external, that the system signals as worthy of further attention and processing. The SN determines the salience of a stimulus, based on input from various other systems, including the dopaminergic reward and LC‐NE systems that we referred to earlier (McCutcheon et al., 2019).

The AIC and ACC, as components of the SN, have both received extensive attention in brain research; therefore, much is known about their basic functions. The insula, in general, presumably plays an important role in integrating information from one's ‘internal environment’ (Craig, 2014), such as energy level, pain, emotions and sympathetic versus parasympathetic activation (i.e. whether one is stressed or not). Those characteristics of the AIC make it a suitable candidate as one of the fundaments of self‐awareness (Craig, 2014).

The other main component of the SN, the ACC has traditionally been linked to performance monitoring, which implies that it compares ongoing actions and outcomes with the direction of one's goals (Carter et al., 1998). In cooperation with other brain structures, such as the nucleus accumbens (part of the dopaminergic reward system), the ACC supports decisions on whether one is willing to spend effort in order to obtain a specific goal (Hauber & Sommer, 2009).

#### 4.3.2 The SN in flow

Given the performance monitoring properties of the SN and the fact that it is considered to act as a switch between the DMN and the CEN, it can be hypothesized to play a role in flow. Specifically, in situations in which there is a match between the task at hand and a person's skills or abilities, the SN may signal that everything is still going ‘according to plan’ and that one is gradually working towards achieving one's goals. This particular configuration of the SN may be one of the building blocks of the subjective sense of control that presumably is an important dimension of flow (see Section 2). In addition, the performance monitoring role of the SN seems to fit well with the notion that flow requires feedback on how one is doing (see Section 2). The SN may particularly provide process feedback information and translates that into consciousness knowledge regarding whether performance is still optimal or not (Figure 4).

If, during task engagement, the skills/challenge balance is disturbed, because, for example, the task becomes more difficult, performance errors start to occur which are registered by the SN. In that case, two things may happen. The first one is that the increase in errors indicates that the task has become more challenging and that more effort or focus is required. Subsequently, the CEN may become more active, the DMN may become even less active and one's flow experience gets more intense (more focus, more effort, less awareness of self or surroundings). Such increase in flow intensity when the challenge increases, but is still manageable, has indeed been reported in the literature (e.g. Csikszentmihalyi & Nakamura, 2010).

The second possibility is that the task challenge increases to such an extent that it becomes too difficult, and errors accumulate. When this happens, flow is diminished. The SN will switch the activation pattern from more CEN and less DMN to more DMN and less CEN. In other words, one starts to become more self‐aware again, and gets more easily distracted, the flow‐state has then, in effect, ended.

Given the wide range of functions and tasks in which the SN is involved, many questions are still open about the scope of influence of this network on flow. For example, besides the DMN, the SN may also play a role in the level of self‐awareness. Craig (2014) posed a theory stating that the integration of interoceptive information in the AIC leads to so‐called global emotional moments, which he assumed are basic building blocks of self‐awareness. A global emotional moment can best be understood as an analogy to a frame in a movie. A rapid sequence of frames is perceived as a movie. Similarly, a rapid sequence of global emotional moments in a processing buffer leads to the perception of oneself as an entity in a time, which would be the raw material for consciousness. Craig also proposed that the fluctuations in the number of global emotional moments in the buffer influence fluctuations in levels of self‐awareness as well as in time perception. During periods of intense negative emotions, for instance, there are many global emotional moments, and consequently self‐awareness is high and time seems to pass by very slowly. An example is a car accident in which people often report that they had the idea that they were very aware of themselves (as if watching a movie) and everything seemed to happen in slow‐motion. In flow, however, people have low self‐awareness and subjective time goes by very quickly. In terms of Craig's theory, this would suggest a low number of global emotional moments per time‐unit. The empirical finding that flow is associated with more parasympathetic activity fits with this notion because such finding indicates low levels of stress during flow and therefore, possibly less global emotional moments.

It is not clear yet, how the empirical findings on AIC activation in flow have to be interpreted in light of theories such as the one discussed above. In their MRI experiment, Ulrich et al. (2014) reported bilateral *increased* activation of the AIC during a flow condition. However, the left AIC was more strongly activated than the right AIC. The latter finding seems in accordance with Craig's so‐called asymmetrical emotional processing account of the AIC. This account states that differences between the left and right AIC may underlie that subjective time goes slower during negative emotional situations, but seems to go faster during positive emotional situations. According to Craig, the positive emotional situation would be characterized by a stronger activation of the left AIC compared to the right AIC. This is what might happen during flow.

As the theory of AIC‐driven global emotional moments is relatively new, many questions on how this may relate to flow remain open. Nevertheless, we consider it a potentially relevant theory that can further delineate the role of the SN in flow.

## 5 A NEUROSCIENTIFIC MODEL OF FLOW: SYNOPSIS

So far, we have discussed different large brain systems and networks in detail and elaborated on how they can map onto the various dimensions of flow. In the present section, we will briefly revisit the main ideas from the previous sections in order to show how the different components can form a coherent neuroscientific account of flow. Obviously, given the scope and multifaceted nature of flow, it cannot be expected that such an account can explain *all* aspects of this state or includes *all* brain structures/network involved. Nevertheless, it can provide a general picture on how the brain establishes flow. In addition, although by definition, such a model has to be limited in scope, in our view, providing a broad neuroscientific model of flow is useful and needed because it would allow a more precise investigation of flow and may also lead to new lines of research on the topic.

As a recap, the neuroscientific model of flow starts with the consistent finding that flow is more likely in intrinsically motivating, meaningful or enjoyable tasks (e.g. Csikszentmihalyi & Nakamura, 2010). This suggest that, in light of all the different behavioural options available at any given moment, the brain has, nevertheless, ‘decided’ that it is worthwhile to fully engage in it. Dopaminergic reward and the LC‐NE systems likely play a role in enhanced engagement in the task, together with the almost complete temporary neglect of all other options. When reward systems are active, one is less likely to experience negative or inhibiting mood states such as fatigue, hunger or low expectations. On the contrary, the higher dopaminergic activity will coincide with activating moods and cognitive states such as optimism and energy. Moreover, those rewarding aspects should reinforce the drive to be in a flow, hence its autotelic properties.

There is consistent empirical evidence that a match between a person's skills and the task requirements enhances the probability of experiencing flow (Moneta, 2012; Peifer et al., 2014). The performance monitoring aspects of the SN seem to be a plausible candidate to play a role in this. Components of the SN, that is, the ACC, continuously evaluate feedback and check whether one is still ‘in control’. This maps well onto the flow dimensions of control and feedback, which have been identified in the literature.

Given that being in flow often occurs on tasks that have rewarding elements, the LC‐NE system may ensure that the level of arousal and response‐related processing are optimal. As soon as the challenge becomes too high and things start to break down (e.g. more errors start to occur), the probability of flow being disrupted increases. In that case, the LC‐NE system either switches to a mode that induces a general withdrawal, or to a stress/distractibility mode, which are both incompatible with flow.

During flow, high focus, low self‐awareness and speeding of subjective time occur (Hancock et al., 2019). This set of characteristics can partly be explained by the interaction between the three large networks we discussed, namely the DMN, the CEN and the SN. As a central network in self‐referential thoughts, DMN activation would be low, whereas the CEN, which supports focused attention, would be active in tasks that require concentration. This presumed configuration of networks during flow has been partly supported by several empirical studies using brain scanning (fMRI) or EEG measures (Harmat et al., 2015; Ulrich et al., 2014).

All in all, the neuroscientific account of flow as outlined here has the potential to link specific configurations of brain activity to various well‐known behavioural and subjective dimensions of flow. Moreover, it can provide insight in the neurocognitive mechanism of the dynamics of flow and the conditions under which it is likely to be induced versus disrupted or prevented. Table 2 provides a summary of the various psychophysiological and brain measures and their contributions.

**Table 2:** Overview of psychophysiological measures and their contribution to understanding the neuroscience of flow

Psychophysiological method	General findings/contributions	Selection of references	Potential future contribution
fMRI	Flow experience associated with lowered activation of brain areas associated with the Default Mode Network (DMN), e.g., medial prefrontal cortex Increased activity during flow experience in brain areas associated with top‐down attentional control, e.g., dorsolateral prefrontal cortex, left inferior frontal gyrus Indications of flow experience‐related activity of dopaminergic brain reward systems, e.g., increased putamen activity Reduced amygdala activation during flow experience (= less anxiety/stress)	Ulrich et al. (2014), Ulrich et al. (2016)	Examining activation of the LC‐NE system during flow experience
Structural MRI/PET	More Gray matter volume in flow‐prone individuals Higher density of D2 dopamine receptors in striatum in flow‐prone individuals	Harmat et al. (2015), Manzano et al. (2013)	Discovering the structural brain differences related to the flow proneness
fNIRS	No relationship between flow experience and frontal cortex oxygenation	Harmat et al. (2015)	Testing brain activity during various activities that can induce flow. Also particularly suited for testing flow during social interaction.
EEG	Lower alpha power and increased theta power associated with flow experience	Katahira et al. (2018)	Dynamic EEG assessment during flow in real‐life (ecologically valid) situations
Cardiovascular measures	Higher respiratory depth during flow experience indicating more relaxation and parasympathetic involvement Flow experience is associated with moderate levels of arousal	Peifer et al. (2014)	Revealing the role of effort (e.g. heart rate variability) during flow

Abbreviations: (f)MRI, (functional) Magnetic Resonance imaging; PET, positron emission tomography; fNIRS, Functional near‐infrared spectroscopy; EEG, Electroencephalography.

## 6 A NEUROSCIENTIFIC MODEL OF FLOW AS A COMPASS FOR FUTURE RESEARCH?

Beyond direct insight into the underlying mechanisms of flow, another presumed advantage of a neuroscientific flow model is that it can guide future research and can lead to new research questions that would not be directly obvious from more ‘traditional approaches’ to the topic. Below, we provide examples of such questions.

Many of the functionalities of the brain systems we discussed are very general and are involved not only in flow, but in numerous other tasks and states. Thus, one question is whether there is a specific configuration of networks that is unique for flow, or whether there is nothing specific to flow except that it reflects the extreme point of focused attention. In case of the latter, flow would only be a matter of gradients of attention, and over time, the best strategy might be to assimilate flow in the broader and more general literature on motivation and attention. If, however, flow is associated with a more unique pattern of network states, then it would be useful to identify this pattern more precisely. The present review partly addresses this topic.

There are several possible ways to address the questions mentioned above. Of course, fMRI studies would be able to directly show which brain areas are active or inactive during flow (e.g. Ulrich et al., 2014, 2016). However, inducing flow while participants are in a scanner may be quite challenging and remains somewhat artificial. Alternatives are the use of electromyography (EMG) or electroencephalography (EEG) approaches to study flow in more natural settings (Cheron, 2016). Particularly, the latter method may be promising, as with advancing techniques, EEG equipment is becoming more wearable and less inconvenient for the participants. For example, it would be possible to use such EEG equipment in chess players who engage in actual competitions in which they sometimes experience flow. Also relevant in this context is that several authors have proposed that, in contrast to common use, EEG can not only be used to examine brain activity but can also be used to assess which brain structures are active (Michel & Murray, 2012). Thus, EEG may serve as a brain imagining technique in this context. A good example of such an approach is the study of Leroy and Cheron (2020). They examined EEG in a professional tightrope performer and, compared to more stressful task periods, found distinct patterns of brain activity during flow‐like periods.

Another open question that may complicate the search for a neurological profile of flow and that has received little attention in the literature so far is whether there are ‘different states of flow’ that partly depend on the task requirements. For example, the flow that occurs during sports (e.g. the runner's high) may be different from the one experienced during solving complex problems, which may, again, be different from the flow that one experiences during highly engaging social interaction. In each of these situations, different network activation patterns may be present. DMN activation, for instance, indicative for self‐referential thinking, may be low during most flow experiences, but not necessarily low during flow related to social interaction. Specifically, several studies now showed that the DMN is not only active during resting states (e.g. day dreaming and thinking about oneself) but also during social tasks (Elton & Gao, 2015; Schilbach et al., 2008).

A third example of a research question regarding the nature of flow that has been difficult to address with more traditional approaches is what mood states are experienced during the actual flow. In many conceptualizations and operationalizations of flow, it is assumed that enjoyment is a key component (Csikszentmihalyi, 2014). However, this assumption is mainly based on survey research in which participants afterwards answer questions on how they felt during their flow. One possibility is that the optimal performance during flow leads to satisfaction, happiness and enjoyment *after* the event, which then might be wrongly attributed to the mood state *during* the flow. Consider, for instance, an athlete competing with others. During the competition, the athlete may be in a flow and near the end gives everything to win. Enjoyment or happiness may not be the best way to describe the mood the athlete is feeling during such a struggle. Once the competition is over and the athlete has won, however, he or she will feel very satisfied with the performance and, looking back, reports having enjoyed the competition. A neurological and psychophysiological approach may be useful here. For example, to examine whether there are any neuroscientific indications of good mood during flow. The involvement of the reward system, as referred to earlier, does seem to suggest that flow may be associated with positive mood states. On the other hand, reward systems may mainly be a more accurate indication of activating emotions instead of positive emotions per se.

## 7 CONCLUDING REMARKS

Several decades of research confirmed that flow is associated with optimal performance and with mental health benefits, including a better mood and sense of meaningfulness (Csikszentmihalyi, 2014; Demerouti, 2006; Fullagar & Kelloway, 2009). Accordingly, the relevance of flow for human performance and well‐being has been widely acknowledged. Despite this accumulation of knowledge on flow, neuroscientific research on the topic remains surprisingly sparse and there is no consensus yet about the neurological processes involved. In the present review, we aim to contribute to this field by discussing various large‐scale candidate brain systems whose broad functionalities may explain the manifestations of flow. Such an account is highly needed if we want to advance insight in what is flow, and also what is not flow. Including explanations at the neurological level allows a more detailed and precise scrutiny of flow and is needed to strengthen the position of the phenomenon in the literature.

## CONFLICTS OF INTEREST

The authors wish to declare that they do not have any conflict of interests.

## AUTHOR CONTRIBUTIONS

DvdL, MT and AAB all contributed to the ideas and writing in this paper.

### Peer Review

The peer review history for this article is available at https://publons.com/publon/10.1111/ejn.15014.

## Data Availability

Not applicable (this article reports no primary data).
